# Current and Emerging Therapy for Celiac Disease

**DOI:** 10.3389/fmed.2014.00006

**Published:** 2014-03-24

**Authors:** Govind K. Makharia

**Affiliations:** ^1^Department of Gastroenterology and Human Nutrition, All India Institute of Medical Sciences, New Delhi, India

**Keywords:** zonulin antagonist, gluten-free, HLA-blockers, transglutaminase inhibitor, probiotics, endopeptidases, glucocorticoids, ancient wheat

## Abstract

At present, strict and lifelong gluten-free diet is the only effective treatment for celiac disease. Even small amounts of gluten (50 mg/day) can be immunogenic; therefore all food and food items and drugs that contain gluten and its derivatives must be eliminated completely from the diet. While prescribing gluten-free diet is easy; the key to the success is the dietary counseling by a nutrition specialist and maintenance of adherence to GFD by the patient. In recent times, a number of targets to halt the process of immunological injury have been explored to find out alternative treatment for celiac disease. These targets include exploration of ancient wheat if they are less immunogenic, intra-luminal digestion of gluten using prolylendopeptidases, pretreatment of whole gluten with bacterial-derived peptidase before ingestion; prevention of passage of immunogenic peptides through the tight junctions such as zonulin antagonists, Blocking of HLA-DQ2 to prevent binding of immunogenic peptides, inhibition of transglutaminase 2, immune-modulation, and induction of tolerance to gluten using gluten tolerizing vaccines, use of gluten-sequestering polymers, use of anti-inflammatory drugs (glucocorticoids, budesonides) and anti-cytokines such as anti TNF-α, and anti-interleukin-15. While many of these targets are still in the pre-clinical phase, some of them including zonulin antagonist and endopeptidases have already reached phase II and phase III clinical trials. Furthermore, while these targets appear very exciting; they at best are likely to be used as adjunctive therapy rather than a complete replacement for gluten-free diet.

## Introduction

At present, strict and lifelong gluten-free diet is the only effective treatment for celiac disease (1). Even small amounts of gluten (50 mg/day) can be immunogenic; therefore all food and food items and drugs that contain gluten and its derivatives must be eliminated from the diet completely (2). While prescribing gluten-free diet is easy; the key to the success is the dietary education by a nutrition specialist and maintenance of adherence by the patient (3). Patients should be encouraged to join a celiac disease support group, because members of the support group are generally more knowledgeable and adhere to their diet better than non-members. The support group provides platform to discuss and share many diet related issues and they learn from the experience of the other members. The overall target of treatment of celiac disease are summarized in Box 1.

Box 1 **The overall target of treatment of celiac disease.**

**Induction of remission**

Clinical:
  Resolution of symptoms
  Improvement in the quality of life
Serological: Normalization of the titer of anti-celiac antibodies
Histological: Reversal of histological abnormalities
Avoidance of long-term complications

**Maintenance of remission by continuation of gluten-free diet**

**Screening of the family members for celiac disease**

**Secondary prevention**

Early detection of diseases and early institution of treatment

## Evolution of Dietary Management of Celiac Disease

Dietary management was a mainstay of treatment of celiac disease even in the early part of the twentieth century (4). During 1930s, clinical improvement was observed with several differing diets including a Dutch mussels diet suggested by Gee and the banana diet popularized by Haas (5). Stools of such patients were quiet greasy and worsening of their diarrhea after a carbohydrate diet led to another dietary approaches such as reduction or almost complete elimination of dietary fat or carbohydrates. During that time, the mortality of patients with celiac disease was very high.

Then came a remarkable observation by a Dutch pediatrician Willem Dicke who gave the birth to an idea from listening to one of his child patients’ mothers. The mother of the patient told Willem Dicke that her child used to become better if he did not eat wheat products. From a clinical observation of one child and through years of historical questioning and empirical dietary therapy, he concluded that wheat was the toxic agent leading to celiac disease (6). He then wrote his thesis on a remarkably careful series of clinical studies, which resulted in basic understanding of the toxic effects of wheat protein in celiac disease (7). We can only admire and learn from such great physicians with superb clinical acumen whose observations led to great discoveries. Toward the end of World War II, the so-called “winter of starvation” when even bread was not available in Holland; children with celiac disease paradoxically improved even though they were consuming a starvation diet (almost devoid of wheat products). When bread was airdropped in Holland, these children rapidly deteriorated. Such an observation further strengthened the idea that ingredients of wheat were toxic agent for celiac disease.

Having initially established that removal of dietary whole wheat products regularly improved symptoms and growth retardation, Dicke studied the effect on his patients of other grains and wheat components including rye flour, wheat flour, wheat starch, rice flour, and cornstarch, each for several weeks at a time. Following the observations of the effect of different diets in celiac disease patients, Dicke published his work in 1953 (7). Since >40% of amino-acid contents of gliadin was glutamine, the demonstration that deamidation of gliadin with hydrochloric acid and subsequently and use of deamidated gliadin abolishing the ill effects of gliadin further confirmed that, gliadin fraction of wheat was possibly the pathogenic component (8). This was how, it was established that gluten is the pathogenic agent for celiac disease.

## What is GFD?

The philosophy behind the GFD has varied across the world according to the strictness of what is considered “gluten-free” (9). In Australia and New Zealand, a “no-detectable gluten” diet is taught. This defines a gluten-free food as one in which gluten cannot be detected by the most sensitive validated assay and equates to about 2–5 ppm. In 2012, the level of gluten in foods that could be considered gluten-free was redefined as 20 ppm (mg/kg), with “very low gluten” foods defined as those <100 ppm (10).

## How Much Gluten is Toxic?

Gluten intake varies from population to population and depends upon dietary practices. A typical North Indian diet, where flat bread is the usual meal, contains about 25–30 g gluten per day; whereas average gluten intake in the West varies from 10 to 20 g/day. In a double blind, placebo-controlled prospective study; Catassi et al. (2) demonstrated that an intake of as little as 50 mg of gluten per day for 3 months was sufficient to cause a significant decrease in the intestinal mucosal villous height/crypt depth ratio. Furthermore, a daily intake of gluten of lower than 10 mg is unlikely to produce significant histological abnormalities (11). The gluten-free diet with the threshold at <20 ppm of gluten ensures an intake of <50 mg/day and provides a sufficient safety margin (12).

## Gluten is Ubiquitous in Food Products

Gluten is responsible for most of the visco-elastic properties of wheat flour doughs, which helps in making good breads. Because of its visco-elastic properties, gluten is used extensively in the food industry. In fact, efforts have been made to increase the gluten content of the wheat by many wheat scientists. Globally, gluten industry is quiet large and there is a big market for gluten. It may be surprising to know that gluten is present in daily use item such as lipsticks, postage stamps, beer, ice-creams, sweets, confectionary, and many more (13).

With such widespread use of gluten in food items, it is therefore extremely difficult to avoid gluten ingestion completely. Because of lack of gluten labeling on the food items, it becomes almost impossible to know if the particular product has gluten or not. Almost half of patients with celiac disease continue to have active disease despite following gluten-free diet (14). We also observed that most of the patients continue to have varying grade of villous atrophy despite continuing on gluten-free diet from 1 to 4 years (15).

The management of celiac disease is truely different and unique from the treatment of other medical or surgical diseases. The center stage of treatment of celiac disease is dietary counseling of the patient and the family by a nutrition specialist and a regular reinforcement for adherence. The dietary councilor should have sufficient knowledge about the food and food products. It is not only about prescribing gluten-free diet but also is to provide an individual patient specific well balanced diet. There are not enough trained nutrition specialists in India. While a physician can diagnose the disease; he/she may not have sufficient knowledge or time to explain to the patient about gluten-free diet; and therefore a nutrition specialist/dietician must be involved in the management of such patients.

## Response to GFD

Most patients with celiac disease respond to gluten-free diet within weeks to months. Patients first start feeling an overall wellbeing; symptoms of anemia, nutritional deficiencies start improving within weeks. The normalization of hemoglobin and complete recovery from diarrhea might take months. The adolescent and children starts gaining their height within months. There is a progressive decrease in the titer of celiac related antibodies. While mucosal healing is the ultimate goal of the therapy, the complete healing of the mucosa is achieved in a minority of adults although it is usual in children (11, 16).

## Adherence to GFD

Wheat is consumed worldwide and gluten is constituents of diet in many and varied forms. Since gluten is ubiquitous and used extensively in the food industry, many food items may be contaminated with gluten (17). Even the homemade food may get contaminated with traces or even moderate amount of gluten (18). Furthermore, even a small amount of gluten is immunogenic to the susceptible host. Maintenance of gluten-free diet is quiet demanding and requires considerable amount of motivation by the patient and the family. There is, thus, a need to develop alterative/adjuvant therapies for the patients with celiac disease.

Patients with celiac disease diagnosed in their early part of life comply better to GFD regimen than those who are diagnosed in the adulthood. The compliance to GFD is generally poor in those who have minimal symptoms or are diagnosed on screening than those who have overt symptoms (19). Adherence to a gluten-free diet is improved in individuals with a thorough understanding of the diet and also those who participate in a celiac disease advocacy group (20). As the patient grows from childhood to adulthood and become asymptomatic, the compliance gets worse in adulthood. The reasons for non-compliance are poor palatability, cost, availability, and social pressures (21). Non-appearance of acute symptoms after inadvertent or deliberate ingestion of gluten might make the patient more confident and induce them to try gluten at other occasions also. Many patients in remission might try gluten to see what happens to them with gluten intake.

We need to emphasize to patients that symptoms and disease are two different aspects. Disease might have to be advanced before symptom arises and most patients may not develop symptoms with occasional ingestion of gluten. Even occasional intake of gluten in patients with celiac disease can maintain the immunological cascade of events and the inflammation in the intestine. The nature and natural history of persistence of low-grade inflammation is not known at the present time.

Patients on gluten-free diet may have significant negative impact on their quality of life. They may feel socially retracted. Furthermore, these patients have produce considerable psychological, emotional, and economic stress (22). Furthermore, non-availability of GFD, while traveling, make them avoid both professional and social commitments.

A small group of patients with celiac disease (2–5%) fail to improve clinically and histologically upon elimination of dietary gluten. This complication is referred to as refractory CD, and it imposes a serious risk for developing lethal enteropathy-associated T cell lymphoma (23).

## Non-Availability of Gluten-Free Food Products

The families of patients with celiac disease can prepare gluten-free food at home by learning the ways to avoid gluten contamination. Furthermore, gluten-free food can be made available commercially. Commercially available GFD are of two types, those having limited shelf-life (gluten-free breads, biscuits, cakes, etc.), and others having a relatively longer shelf-life. At present, gluten-free products are not widely and freely available; they are expensive and have poor shelf-life. There is a limited availability of gluten-free products in India. There is an urgent need for gluten-free foods in India and both food technology institutions and the food industry should get involved.

## Need for an Alternative Treatment for Celiac Disease

Since gluten is ubiquitous and used extensively in the food industry, many food items may be contaminated with gluten. Even the homemade food may get contaminated with traces or even moderate amount of gluten. We should remember that even a small amount of gluten is immunogenic to the susceptible host. Maintenance of GFD is quiet demanding and requires considerable amount of motivation by the patient and the family. There is thus a need to develop alterative/adjuvant therapies for the patients with celiac disease.

The gluten ingestion varies from population to population. While most people in the west ingest about 13–15 g of gluten per day; the gluten ingestion is higher especially in the Northern part of India and averages about 20–30 g gluten per day (24). Such large dose of gluten in a normal diet is less likely to be neutralized by any form of alternative therapy. Therefore, one should realize that GFD is the best and ideal for patients with celiac disease. The potential alternative targets, which are presently investigated, are expected to neutralize only a small amount of gluten exposure (say 1–3 g of gluten per day) rather than 10–30 g of gluten. Therefore, such agents will protect patients from minor unintentional or unavoidable gluten ingestion rather than complete neutralization of total gluten intake of a normal individual.

## Emerging Therapy for Celiac Disease

There are many targets in the inflammatory cascade of events, which can be modulated in order to halt the process of immunological injury (25–30).

## Approach 1: Reduction in the Exposure of Gluten

### Use of ancient wheat

One of the approaches to decrease exposure to immunogenic gluten in genetically susceptible person is to grow genetically modified grains, which are devoid of immunogenic epitopes (24, 25, 31). Although it looks very attractive, such an approach however is very challenging. Gluten contains a large number of immunogenic peptide, which can induce immune system in genetically susceptible individuals (32). The genes coding for these immunogenic peptides are located in different genetic loci in the wheat genome, therefore gene silencing or gene deletion do not appear to be the viable options.

Over the past five decades, several changes in the pattern of wheat consumption has been observed including an increase in per capita consumption of wheat, an increase in the use of gluten in food processing and an increase in the consumption of processed foods. Furthermore, an increase in celiac disease-related T cell stimulatory epitopes has also been observed in the wheat. It is conceivable that these changes in the wheat consumption pattern and increase in T cells stimulatory epitopes in wheat may be the reasons for an increase incidence of celiac disease world over. It is be thus important to understand a bit about the genetics of wheat and its immunogenic peptides involved in celiac disease (33).

In the evolutionary process, the genome of wheat has changed from diploid (14 chromosomes) to hexaploid genome (42 chromosomes) (34). The genome of the most ancient wheat is diploid and is called AA, BB, DD. These grass-like wheat species had a very low seed yield and their seed dropped easily. Natural hybridization between two of these diploid species led to birth of the tetraploid *Triticum* species having AABB genome. Finally, around 4000 BC, natural hybridization between tetraploid (AABB) species and diploid species (DD) led to origin of hexaploid wheat species. Bread wheat (*Triticum aestivum*), the wheat used presently all over the world, is an allohexaploid species resulting from natural hybridization between a tetraploid *T. turgidum* (*dicoccum*) carrying the AABB genome and a wild diploid species *Aegilops tauschii* carrying the D-genome. The introduction of the D-genome in the wheat improved the bread-making properties of the wheat (35, 36).

Most commonly used wheat at the present time is hexaploid wheat (*T. aestivum*); and tetraploid wheat (Durum wheat) is used sparingly. The immunogenicity of gluten peptides present in various wheat varieties having diploid, tetraploid, or hexaploid genome is different. Even amongst thousands of different *Triticum* wheat varieties/accessions (hexaploid), the immunogenic potential may vary. These facts raised a question, if there is existence of safer/less immunogenic wheat varieties for patients with celiac disease.

The gluten digest from the selected cultivars of *Triticum tauschii*, the DD genome donor, contains all the T cell epitopes identified to be immunogenic in patients with celiac disease. Furthermore, isolation and characterization of intestinal T cells from celiac disease patients have revealed several distinct but similar DQ2 and DQ8 epitopes. These epitopes are generally very rich in proline and glutamine residues. The best known and most immune-competent peptide for HLA-DQ2^+^ celiac disease patients is a 33-mer peptide encompassing 57–89 N-terminal region of certain α-gliadin proteins. This 33-mer peptide is resistant to gastrointestinal proteolysis, does not require intra-cellular processing for presentation, and binds efficiently to HLA-DQ2 (37). Gluten, from the wheat having AA genome and most likely also BB genome, lacks sequences equivalent to the 33-mer immunogenic peptide fragment. Experiments with mutant wheat lines suggest that most immunogenic peptide sequences are encoded exclusively by γ-gliadin genes located on chromosome 6D (38). This explains why some durum wheat (AABB) cultivars do not have these immunogenic peptides.

Overall the present set of evidences tends to suggest that the ancient wheat cultivars are less immunogenic for patients with celiac disease (39). Attempts to detoxify the gluten epitopes by a number of different methods are being taken. Genetic deletion of certain gliadin genes, notably the complete deletion of α-gliadin in Chinese spring wheat, led to the decrease in the number of T cell-activating epitopes without affecting the baking quality of the wheat flour dough (40).

In fact, there are now efforts being made to decrease the immunogenic gliadin peptides in wheat using RNA interference technology (41).

There are many challenges in the process of the modification of gluten contents in the wheat varieties and some of them are: (i) the modifications in the protein contents may lead to loss and decrease in baking characteristics, the main property of wheat, which makes it a preferred cereal by the humans; (ii) all the immunogenic peptides in wheat are still not known; and (iii) there is a potential for contamination (if sown in nearby fields) of the future genetically modified grains with the wild strains during cultivation, pollination, harvesting, and processing.

## Approach 2: Assisted Digestion of Gluten

The principal of assisted digestion is to detoxify the gluten either in the gastrointestinal lumen using endopeptidases (Intra-luminal enzymatic digestion) or using predigested gluten where gluten is digested partially before ingestion.

### Intra-luminal enzymatic therapy

Dietary proteins reaching the intestinal lumen are digested by gastric pepsin and pancreatic proteases and further degraded by brush border enzymes (dipeptidases, tripeptidases) into amino-acid, dipeptides, or tripeptides, which are transported across the enterocytes. The principal toxic components of gluten are proline- and glutamine-rich peptides that are resistant to proteolysis by gastric, pancreatic, and intestinal brush border membrane enzymes (42). Human do not have enzyme to break the bond between proline and glutamine. Therefore, gluten protein is not digested completely and partial digestion of gluten leads to creation of multiple peptides, which induce immune system in genetically predisposed people and cause celiac disease (43).

Therefore, one of the logical approaches is to use enzyme(s) that can break/digest the bond between proline and glutamine in the gluten (25, 26, 28). Prolyl endopeptidases (PEPs) can break bonds between proline and glutamine and can be derived from microorganisms such as *Flavobacterium meningosepticum*, and *Sphingomonas capsulate* (44). Most of these enzymes however, are irreversibly inactivated in the stomach by pepsin and acidic pH, and do not reach in the intestine in adequate concentration, and thus require to be delivered in acid resistant capsule forms.

A combination of endoprotease B2, a glutamine-specific protease from germinating barley, and PEP from *S. capsulata* could efficiently break down whole wheat gluten *in vitro* and in a rat model *in vivo*, and abrogated largely its immunogenic potential as assessed with several gluten-specific T cell lines (45–49). While glutamine-specific endoproteases (EP-B2) hydrolyzed extensively the gluten network present in the bread into relatively short (but still inflammatory) oligopeptides; prolyl specific endopeptidase from *S. capsulata* rapidly detoxified the oligopeptides after primary proteolysis at internal proline residue level to yield non-toxic metabolites (50). Both these enzymes are active and remain stable at acid pH and can therefore be administered as lyophilized powders or simple capsules or tablets (51).

ALV003 (developed by Alvine Pharmaceuticals) is a combination of two different gluten-targeting proteases (glutenases) with complementary substrates. It contains a cysteine-endoprotease derived from germinated barley seeds and a PEP from *S. capsulata*. Both of them are active in acidic medium and their glutenase activity is maximized in a 1:1 formulation. In a Phase 1 clinical trial, 20 patients with celiac disease in remission were randomized to receive a diet containing gluten (16 g/day for 3 days) pretreated with ALV003 and a diet containing gluten and placebo. Patients who received ALV003 gluten had significantly lower immunological activation compared to the group that received placebo (52). A subsequent phase 1b study showed that ALV003 was very effective, in the stomach, in breaking down food with high gluten content (53).

In a double blind, placebo-controlled phase 2a clinical trial on 41 adults with celiac disease, on GFD for one or more years, were randomized to ALV003 or a placebo each day for 6 weeks along with 2 g of gluten in the form of bread crumbs. The small intestinal mucosal injury, as measured by villous height/crypt depth ratio, was significantly less in patients treated with ALV003 than those treated with placebo (*p* = 0.0133). Intraepithelial lymphocytes including CD3^+^ and CD3^+^ alpha/beta and gamma/delta subsets, which measure inflammatory response, were essentially unchanged in the ALV003-treated patients but significantly increased in the placebo-treated patients. No serious adverse events were reported (54). Based on the encouraging preliminary results on safety and efficacy at least in short terms; phase III study is being planned using ALV003 as an adjunct therapy with GFD.

There are other sources for PEP and PEP derived from *Aspergillus niger* has also been shown to degrade gluten peptides (55, 56). Furthermore, PEP from *A. niger* (AN-PEP) is derived from the food grade microorganism and is available on an industrial scale. In a cross-over, randomized, double blind, placebo-controlled clinical trial, AN-PEP’s showed ability to detoxify 8 g of gluten contained in a commercial food product when tested in patients with celiac disease in remission (57).

Complete avoidance of gluten is very difficult since gluten is so widely used in food and food products. While, enzyme therapy can protect patients with celiac disease from unwanted or hidden gluten or unwanted exposure to gluten but will not be effective in digestion of normal dietary gluten intake.

### Pretreatment of whole gluten with bacterial-derived peptidase before ingestion

An alternative approach is to detoxify gluten by digestion of wheat gluten and its peptides using bacteria-derived peptidases during food processing. A few selected sourdough lactic acid bacteria are used widely for fermentation of food in the food industry (58). Certain selected lactobacilli added to sourdough for fermentation have been shown to lyse the proline/glutamine-rich gluten peptides reducing their immunotoxicity (59, 60). Fermentation of the wheat with sourdough *lactobacilli led to* decrease in the concentration of gluten to below 10 ppm as shown by Rizello (61). Furthermore, a mixture of fermented wheat flour along with oat, millet, and buckwheat, have been found to retain its baking characteristics. Wheat flour-fermented with selected lactobacilli and fungal proteases (routinely used in bakeries) led to decrease in gluten content to <10 ppm (gluten-free). Eight patients with celiac disease in remission were enrolled for the clinical challenge, and they consumed 200 g/day of sweet baked goods equivalent to 10 g of native gluten. One patient interrupted the trial after 15 days and another after 30 days due to difficulties in the adherence to daily consumption. None of the patients showed worsening in hematological, serological parameters, and intestinal permeability during 60 days of challenge. This study showed that a wheat flour-fermented product, having gluten completely degraded, is not toxic for patients with celiac disease (62).

In another study from Italy, patients were randomly assigned to consumption of 200 g/day of natural flour baked goods (NFBG) (80,127 ppm gluten; *n* = 6), extensively hydrolyzed flour baked goods (S1BG) (2480 ppm residual gluten; *n* = 2), or fully hydrolyzed baked goods (S2BG) (8 ppm residual gluten; *n* = 5) for 60 days. Two of the six patients who consumed NFBG discontinued the challenge because of symptoms; all had increased levels of anti-tissue transglutaminase antibodies and small bowel deterioration. The two patients who ate the S1BG goods had no clinical complaints but developed subtotal atrophy. The five patients who ate the S2BG had no clinical complaints; their levels of anti-tTG antibodies did not increase, and their Marsh grades of small intestinal mucosa did not change (63).

Among probiotic preparations, VSL#3, a highly concentrated mixture of lactic acid and bifidobacteria, was able to hydrolyze completely the α2-gliadin derived epitopes 62–75 and 33-mer peptide (64). Furthermore, probiotics have also been shown to directly modulate the epithelial cells. Some probiotic strains, including the VSL#3 preparation, increase the epithelial barrier function by stabilizing tight junctions (65, 66).

## Approach 3: Prevention of Passage of Immunogenic Peptides through the Tight Junctions

Tight junctions regulate the passage of molecules through intercellular spaces between epithelial cells (paracellular transport). Intercellular junctions are composed of tight junctions (most apical), adherens junctions, gap junctions, and desmosomes. Tight junctions are regulated by a number of cytoskeletal proteins including trans-membrane proteins (claudin family, occludin, junctional adhesion molecules) and cytoplasmic protein (ZO-1, 2, 3) (67). In normal conditions, harmful bacteria and dietary antigens are prevented from passing through the tight junctions. The function of tight junctions is assessed using intestinal permeability (68).

Changes in the paracellular permeability have been hypothesized to be an early event in the pathogenesis of celiac disease (24). The immune system of susceptible individuals gets exposed to the immuno-stimulatory gluten peptides, which passes through the paracellular route (69). Human protein Zonulin, a precursor of prehaptoglobin-2, has been found to be a regulator of epithelial permeability and is highly expressed in the mucosa and blood of patients with celiac disease (70). Zonulin, which is similar to a protein zonula occludens toxin (ZOT) expressed by *Vibrio cholerae*, impairs epithelial tight junctions integrity. A recent study has shown that gliadin binds to the chemokine receptor CXCR3 releasing Zonulin and subsequently increases the intestinal permeability via the MyD88 dependent pathway (71).

Larazotide acetate (AT-1001, developed by Alba Therapeutics, USA) is an octapeptide derived from a cholera toxin, ZOT and has been found to antagonize zonulin via receptor blockade (72, 73). Larazotide acetate is expected to reduce the paracellular transport caused by gluten and ameliorate the activation of the pathological immune cascade. Six clinical trials have been completed with larazotide acetate. Three of them were Phase 1 trials: two trials in healthy individuals (one single-dose and other multiple dose safety studies) and a third safety study in subjects with celiac disease. In all these studies, the safety profile of larazotide was comparable to that of the placebo (23).

Subsequently, three Phase II studies have been completed. In a Phase IIa randomized, placebo-controlled double blind trial; 86 patients with celiac disease in remission were given gluten challenge of 800 mg 15 min after administration of AT-1001 or placebo, three times a day for 2 weeks. While the primary endpoint of improvement in intestinal paracellular permeability was not met; the symptoms triggered by the 2-week gluten challenge were significantly ameliorated by larazotide acetate (72). In the Phase IIb study (randomized, placebo-controlled, double blind), different doses of larazotide (1, 4, and 8 mg) were compared against placebo. The study enrolled 184 patients with celiac disease in remission and were subjected to a 6-week gluten challenge of 900 mg three times a day. Again, larazotide acetate could not demonstrate statistically significant efficacy in the reduction in intestinal permeability, though a favorable trend was observed (74). A new large (320 patients) Phase 2b study is on the way to study different doses (0.5, 1, and 2 mg/day) of larazotide acetate in patients with celiac disease who are non-responsive to a GFD (ClinicalTrials.gov identifier: NCT01396213).

Increase in intestinal permeability is also dependent on Rho kinase (ROCK) activity (75). In addition to regulating tight junction structure and function, Rho A and ROCK are known to regulate axon growth. The benefit of blocking Rho A and ROCK have been well documented for the treatment of spinal cord injury (76). For example, fasudil, an inhibitor of ROCK, is an approved drug in Japan for the treatment of cerebral vasospasm. Whereas the side effect of fasudil makes it inappropriate for chronic, long-term use in patients with celiac disease, the drug could be used to establish whether ROCK inhibition can reverse gluten dependent increased in intestinal permeability in patients with celiac disease (25). If so, a number of next generation ROCK inhibitors are under active evaluation for other indications such as asthma, pulmonary hypertension, and multiple sclerosis, and some of these drugs may hold promise as therapeutic agent for celiac disease (25).

## Approach 4: Blocking of HLA-DQ2 to Prevent Binding of Immunogenic Peptides

As discussed in the previous sections, celiac disease is a genetic disease and almost all of them have HLA-DQ2 and -DQ8 haplotype (77). Gluten peptides after passing through the intercellular junctions get deamidated by enzyme transglutaminase 2 (TG2), which are present in the subepithelial basement membrane. The deamidated peptides become negatively charged and these charged particles have affinity to bind to the grooves present on the HLA-DQ2 and/or DQ8 located on the surface of antigen-presenting cells. The antigen-presenting cells then leads to activation of T lymphocytes (adaptive immune response) (24). Therefore, blocking the binding grooves of these HLA molecules by gliadin antagonist peptides could suppress the antigen presentation process and block the further cascade of T cell stimulation.

The crystallographic structure of HLA-DQ2 and its complex with a deamidated gluten peptide has provided important information for the development of antagonist compounds. Amino-acid substitutions within gliadin T cell stimulatory sequences can convert the immunogenic epitope to either an agonist or antagonist. If antagonist, it can block the inflammatory cascade of events. Ellis et al. (78) reported that substitution of alanine amino-acid at key positions (3, 8, and 10) in the immunodominant peptide of the wheat α2-gliadin (residues 62–75) led to abolition of the immunogenicity of the peptide when tested against T cell clones. In addition, a decapeptide (QQPQDAVQPF) obtained from an alcohol-soluble protein fraction of durum wheat was shown to be antagonistic to gluten toxicity *in vitro* (79). An aldehyde functionalized gluten peptide analog has also been designed, which acts both as a tight-binding HLA-DQ2 ligand and a high-affinity reversible inhibitor of tTG2 (80).

While the concept of celiac-specific HLA inhibition is particularly attractive, there are substantial challenges including getting access to the binding groove of the DQ2/DQ8 on antigen-presenting cells and avoidance of rapid degradation of these peptide agents during delivery. Interference with the immunosurveillance functions of HLA class 2 molecules by blocking them in tissues others than intestine is an important point of consideration.

## Approach 5: Inhibition of Transglutaminase 2

Transglutaminase 2 is a ubiquitous multifunctional mammalian protein that catalyzes the formation of intermolecular isopeptide bonds between glutamine and lysine residues of a few selected proteins (81). Its enzymatic activity is allosterically regulated by several factors, including guanine nucleotides, Ca^+2^, and redox potential. In the small intestinal mucosa of celiac disease patients, TG2 deamidates glutamine residues of gluten peptides and create potent T cell epitopes (82). TG2 also induces crosslinking of gluten peptides with matrix proteins, thereby maintain gluten in the tissue and generate complexes that induce an immune response to additional auto-antigens. Therefore, TG2 inhibitors are thought to have promising avenues for celiac disease therapy (25).

At present, there is no successful mouse model for celiac disease. Therefore, most of the potential therapeutic targets are evaluated on simplified biological systems. There are two notable *ex vivo* experiments that suggest that TG2 inhibition have the potential to benefit patients with celiac disease. In the first study, Molberg et al. (83) showed that culture of small intestinal biopsies of celiac disease patients with either TG2 treated (deamidated) or non-TG2 treated (non-deamidated) gluten resulted in the generation of T cell lines that preferentially recognized deamidated gluten peptides rather than non-deamidated gluten peptides. Furthermore, blocking of the activity of endogenous TG2 in the biopsies with cystamine led to reduction in proliferative responses, in more than half of the T cell lines, to deamidated gluten digests compared to non-cystamine treated controls. Taken together, these two results imply that the gluten responsive T cell populations in intestinal biopsies of celiac disease patients are naturally biased toward recognizing deamidated gluten peptides as opposed to non-deamidated peptides. Blockade of the activity of TG 2 by TG2 inhibitors can reduce the proliferative response of gluten-reactive T cells in the intestine.

In another study, Maiuri et al. (84) showed that the 2-[(2-oxopropyl)thio] imidazolium inhibitor, L682777, was able to prevent the *in situ* crosslinking of gluten peptides to endogenous proteins in thin tissue sections taken from both celiac disease patients and controls. More importantly, they showed that incubation of intact celiac small intestinal biopsies with L682777 prevented T cell activation induced by the non-deamidated form of an immunodominant gluten peptide. In contrast, L682777 was ineffective in controlling T cell activation when the biopsies were incubated with the deamidated version of the same peptide. These results suggest that irreversible inhibition of endogenous TG2 in celiac disease patient biopsies can prevent gluten peptide deamidation and, therefore, reduce T cell activation.

## Approach 6: Immune-Modulation and Induction of Tolerance to Gluten

Although gluten is a normal food ingredient however 1% of the world’s population is unable to tolerate peptides of partially digested gluten, which act as auto-antigens. Therefore, another logical and attractive option to reverse the gluten intolerance is to restore the tolerance to ingested gluten. Protein-based desensitization therapy is often used to treat allergic diseases (85). For example, peptide-based vaccination by intra-dermal injection of cat allergen peptides has shown efficacy in the treatment of cat allergy (86).

NexVax2 is a desensitizing or therapeutic “vaccine” and is under development by a biotechnology company ImmuSanT. NexVax2 uses three gluten peptides with the goal of inducing a “tolerogenic” response in patients with celiac disease. NexVax2 has showed efficacy in HLA-DQ2 transgenic mice having gluten-sensitive T cells (87). In a phase 1 study, 40 well-controlled patients with celiac disease were given subcutaneous injections of increasing doses of up to 90 μg of Nexvax 2 in weekly doses for 3 weeks. While there were some gluten-related gastrointestinal side effects, dose escalation could be completed and safety was acceptable. Vaccinated subjects showed presence of IFN-γ-producing anti-gluten T cells (88).

The real efficacy of such a strategy is still to be established. Synthetic peptide vaccines can be developed to include only a finite number of known immunogenic gluten peptides. There are many issues, such as efficacy and long-term safety, are to be established before peptide vaccines are made available for the management of celiac disease.

## Approach 7: Gluten-Sequestering Polymer

Polymeric binders are used in various clinical disorders to sequester toxic compounds in the gastrointestinal tract. Another novel approach to block gluten toxicity using polymeric binder, poly (hydroxyethyl methacrylate-*co*-styrene sulfonate) [P(HEMA-*co*-SS)]. P(HEMA-*co*-SS) has been shown to complex with α-gliadin in a relatively selective fashion (89, 90). The binder counteracts the toxic effects of gliadin on intestinal epithelial cells *in vitro*. Pinier (91) recently reported important findings regarding the *in vivo* biodistribution and therapeutic efficacy of P(HEMA-*co*-SS) when administered orally (single-dose and repeated administration) in a mixture containing whole wheat gluten and other food components to rodents. They showed that P(HEMA-*co*-SS) hindered significantly the formation of major identified immunogenic peptides from wheat gluten (such as QLQPFPQPQLPY and LQPQQPFPQQPQQPFPQ) by gastrointestinal enzymes. P(HEMA-*co*-SS) was shown to also decrease the production of immunogenic peptides from barley.

The mechanism of action of the polymeric binder may be more complex *in vivo*, it is unknown whether similar degree of inhibition of the peptide can be achieved *in vivo* as achieved *in vitro* experiments. Furthermore, polymeric binders may be useful only for the inadvertent or minimal gluten exposure and have potential role as supportive therapy rather than a complete treatment for celiac disease.

## Approach 8: Anti-Inflammatory Drugs

Like inflammatory bowel disease and rheumatoid arthritis, celiac disease is also an inflammatory disease. Several strategies targeting the involved cytokines, chemokines, and chemokine receptors are being developed in relation with celiac disease. While most patients with celiac disease respond to GFD, the scope for anti-inflammatory drugs will be mainly for patients with refractory celiac disease.

### Glucocorticoids

Glucocorticoids are not used for uncomplicated patients with celiac disease and are generally reserved for celiac crisis, gliadin shock, or refractory celiac disease. Reports on the inhibitory effect of steroids on both B and T cell proliferation in *in vitro* studies or their effect on reduction of the lymphokine level released by the cultured cells indicated that they may be effective if given in addition to GFD to patients with celiac disease (92). Wall et al. (93) showed an accelerated improvement in symptoms by addition of prednisone to GFD in patients with celiac disease. In another *in vitro* study, Mitchison et al. (94) had also demonstrated the inhibitory effects of fluticasone propionate on the harmful effects of gluten. In a more recent study on 20 patients with celiac disease, Ciacci et al. (95) have shown a better clinical response in a group of patients treated with a combination of GFD and budesonide for 4 weeks in comparison to those treated with GFD alone.

We conducted a proof of concept randomized control trial to see the effects of short course of prednisolone along with GFD on the markers of apoptosis and anti-apoptosis pathways, as well as on epithelial cell regeneration in patients with celiac disease. Our hypothesis was to see if addition of prednisolone for a short term, accentuates the mucosal recovery. While addition of prednisolone to GFD led to a significant reduction of apoptosis; it also led to decrease in epithelial cell regeneration (96).

### Cytokines and chemokines

#### Anti-interferon-γ and anti TNF-α

The activation of gluten-sensitive CD4^+^ T cells leads to secretion of IFN-γ, which triggers pro-inflammatory responses including activation of metalloproteinases (MMPs) that results in mucosal injury and villous atrophy. In a similar fashion, TNFα also triggers a proteolytic cascade mediated by MMPs secretion from intestinal myofibroblasts and results in intestinal architectural alteration. Therefore, blockade of these cytokines may prevent the activation of proteolytic MMPs and ultimately resume the intestinal hemostasis (24, 28). IFN-γ-blocking antibodies have been shown to prevent damage to healthy intestinal mucosa exposed to the inflammatory cytokines released by the gliadin-specific T cell lines (97). It has also been demonstrated that gliadin-induced-IFN-γ secretion increases gliadin influx through the intestinal barrier. Thus, IFN-γ-blocking agents may stop this vicious cycle (98). In anecdotal cases, monoclonal antibodies against TNF-α (infliximab) has been shown to be beneficial for patients with severe refractory celiac disease and refractory celiac disease (99, 100).

#### Anti-interleukin-15

Interleukin-15 (IL-15), secreted from the intestinal epithelium and antigen-presenting cells in response to gliadin peptides, plays an important role in the underlying innate immune response in the intestinal mucosa of patients with celiac disease. It induces the secretion of epithelial MICA that binds to NKG2D receptor located on the surface of intraepithelial lymphocytes (101, 102). This ligand receptor interaction is enhanced by IL-15, leading to stimulation and proliferation of cytotoxic T lymphocytes that induce epithelial apoptosis and also results in development of refractory celiac disease and its malignant transformation (103). A study on transgenic mouse models with overexpression of IL-15 and consequent development of autoimmune enteropathy demonstrated that blocking antibody against IL-15 was capable of efficiently reversing the intestinal damage (28, 104). IL-15 also prevents apoptosis of the cytotoxic intraepithelial lymphocytes that plays a central role in the refractory celiac disease via the JAK 3/STAT 5 signaling pathway. IL-15 blocking antibodies were able to induce IEL apoptosis and reduce the number of IELs accumulated in the intestinal epithelium of human IL-15 transgenic mouse model (105). Anti-IL-15 human monoclonal antibody has been evaluated in patients with other autoimmune disease such as rheumatoid arthritis, but despite the promising findings, a human study for celiac disease is still awaited.

#### Interleukin-10

Interleukin-10 (IL-10) is an immunoregulatory cytokine in the intestinal tissue and suppresses inflammatory cytokine secretion from TH1 cells. Therefore, IL-10 is considered as a candidate for the treatment of TH1-mediated autoimmune disorders such as inflammatory bowel disease and celiac disease (24, 28). Although IL-10 was shown to suppress gliadin-induced T cell activation in an *ex vivo* study of cultured intestinal biopsies for celiac disease (106), however a pilot study on patients with refractory celiac disease did not show any pharmacological efficacy (107).

#### Lymphocytic recruitment blockade

##### Integrin α4β7 and MAdCAM-1

For migration of lymphocyte to the site of inflammation (intestinal mucosa in case of celiac disease), T cells express integrin (α_4_β_7_), which are required for their binding to mucosal addressin cell adhesion molecule-1 (MAdCAM-1) located on vascular endothelial cells (108). MAdCAM-1 has been shown to be is significantly upregulated in treatment naïve patients with celiac disease. Therefore, lymphocytic recruitment blockade using blockers of integrin (α_4_β_7_ and MAdCAM-1) could be a potential therapeutic target for patients with celiac disease (109). Furthermore, Natalizumab, a monoclonal antibody against α_4_ integrin, has already been shown to be effective in patients with Crohn’s disease (110).

## Future

A better understanding of the pathogenesis of celiac disease and realization that gluten-free food although the best but not completely acceptable has opened the gates for development of newer targeted drugs. The development of drugs from the concept to bench and from bench to clinical use takes many years and requires an extremely large amount of money and resources. The alternative drug development in the field of celiac disease is still in the early stages of drug discovery. In the past few years, a new novel targets have reached Phase I and Phase II trials and larger randomized controlled trials are on the horizon.

### Commercial scope for pipeline drugs for celiac disease

A GlobalData report based on the data and information sourced from proprietary databases, primary and secondary research and in-house analysis suggests that global celiac disease therapeutics market will be of worth $512.3 million in 2017 (111). This market is expected to grow at a compound annual growth rate of 13.9% to reach $664.4 million by 2019. This high growth forecast is primarily attributed to the increased uptake of the pipeline products and increase in diagnosis rates.

## Conflict of Interest Statement

The author declares that the research was conducted in the absence of any commercial or financial relationships that could be construed as a potential conflict of interest.
